# Artificial intelligence in digital pathology: Principles, applications, challenges, and future directions

**DOI:** 10.1016/j.jpi.2026.100749

**Published:** 2026-10-06

**Authors:** Yifei Wang, Leona Odabachian

**Affiliations:** Trinity School of Medicine, Trinity College Road, Ratho Mill, Kingstown, Saint Vincent and the Grenadines

**Keywords:** Artificial intelligence, Digital pathology, Whole-slide imaging, Multimodal models, Clinical applications, Implementation, Regulatory challenges

## Abstract

This article provides an overview of digital pathology and its evolving role in modern diagnostic practice. We begin by comparing conventional microscopy with digital pathology and outlining the major components of the digital pathology workflow. We then explore whole-slide imaging, its imaging modalities, scanner technologies, image storage, and image management systems. We then examine the role of artificial intelligence (AI) in digital pathology, discussing current AI applications and the spectrum of computational approaches, from conventional feature-engineering methods to deep learning and multimodal models. Finally, we review the clinical applications of digital pathology, summarize its implementation across US medical centers, and discuss the technical, regulatory, and operational challenges that must be addressed for broader clinical adoption.

## Highlights

• Reviews principles and workflow of digital pathology.
• Summarizes whole-slide imaging systems, storage, and image management.
• Explores AI from machine learning to multimodal foundation models.
• Highlights clinical applications across diagnostic pathology.
• Discusses barriers and future directions for clinical implementation.

## Graphical abstract

**Figure:**
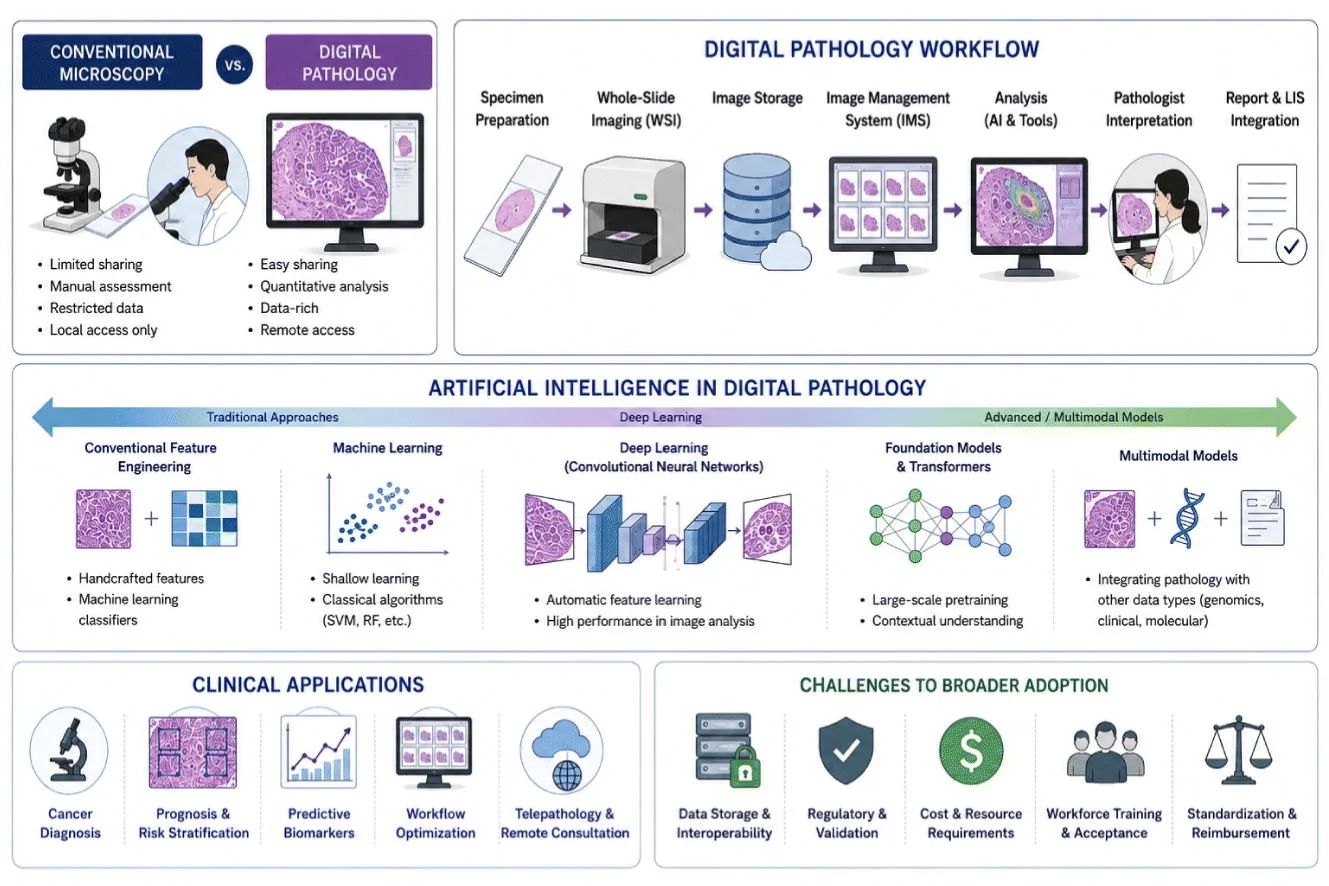

## Digital pathology

### Conventional versus digital pathology

The digitization of histopathology has transformed conventional microscopic diagnosis into a computationally enabled discipline capable of supporting artificial intelligence (AI)-assisted analysis. The traditional pathology workflow relies on the manual examination of glass slides using light microscopy, with diagnosis based primarily on the expertise and visual interpretation of the pathologist. In contrast, digital pathology employs whole-slide imaging (WSI) to convert glass slides into high-resolution digital images that can be archived, shared remotely, and analyzed computationally (Fig. 1).^1^

**Fig. 1:**
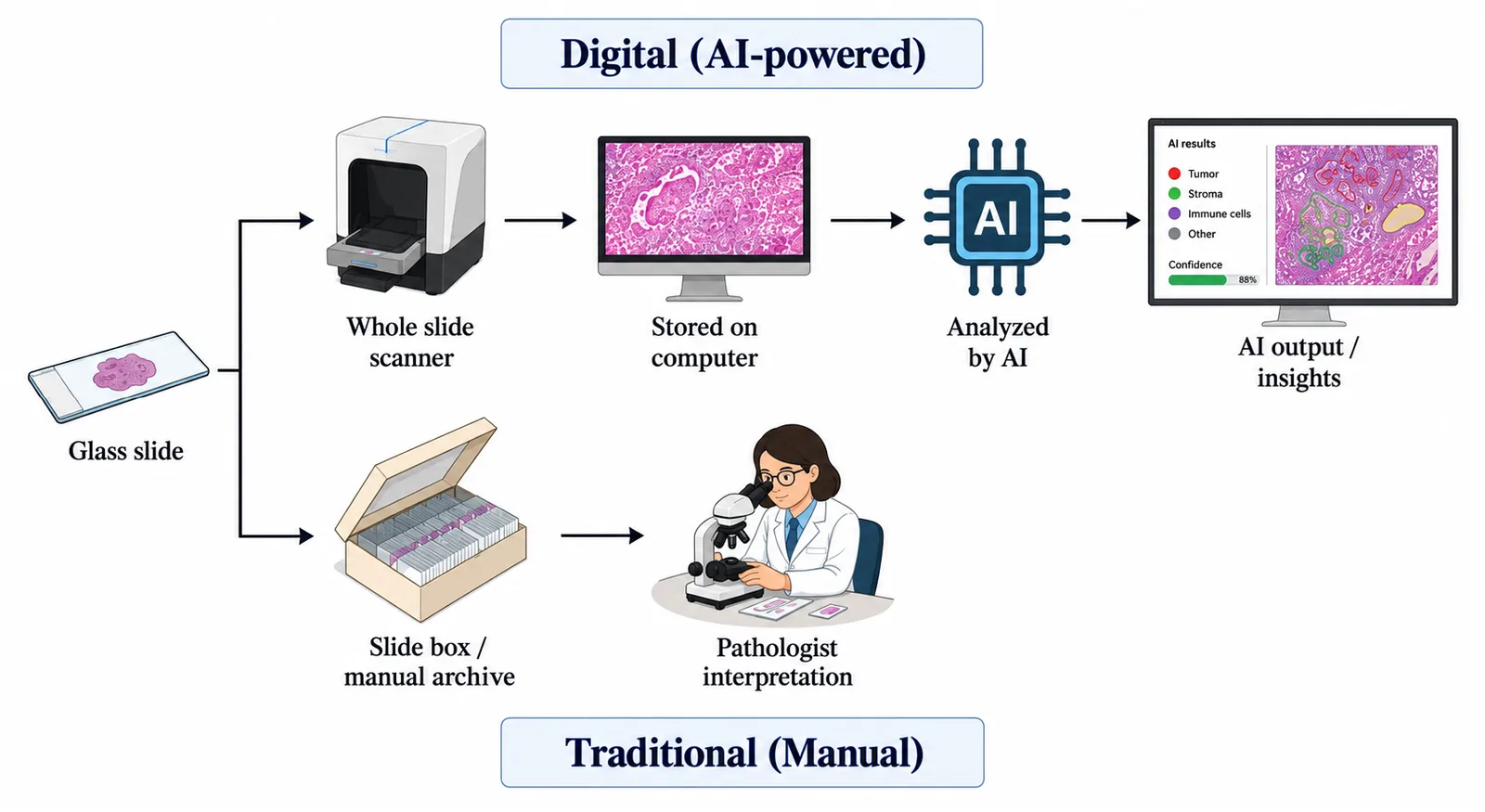
Digital pathology versus traditional pathology. Created in bioRender^2^ and ChatGPT (OpenAI, GPT-5.5).

### Components of the digital pathology workflow

Digital pathology begins with a tissue specimen, which undergoes fixation, processing, embedding, sectioning, and staining to produce a histological glass slide.^3^ Routine slides are typically stained with hematoxylin and eosin (H&E), whereas immunohistochemistry (IHC), special stains, or fluorescence-based assays are performed when clinically indicated.^4^ The stained slide is then digitized using a whole-slide scanner to generate a WSI, which is then stored in a digital image repository and managed through an image management system (IMS). Pathologists review the WSI using dedicated viewing software, with optional AI-assisted image analysis supporting tasks such as detection, quantification, or decision support.^5^ The pathologist's final interpretation is integrated into the lab information system (LIS), which interfaces with the electronic health record (EHR) to make diagnostic results available within the patient's clinical record (Fig. 2).^6^

**Fig. 2:**
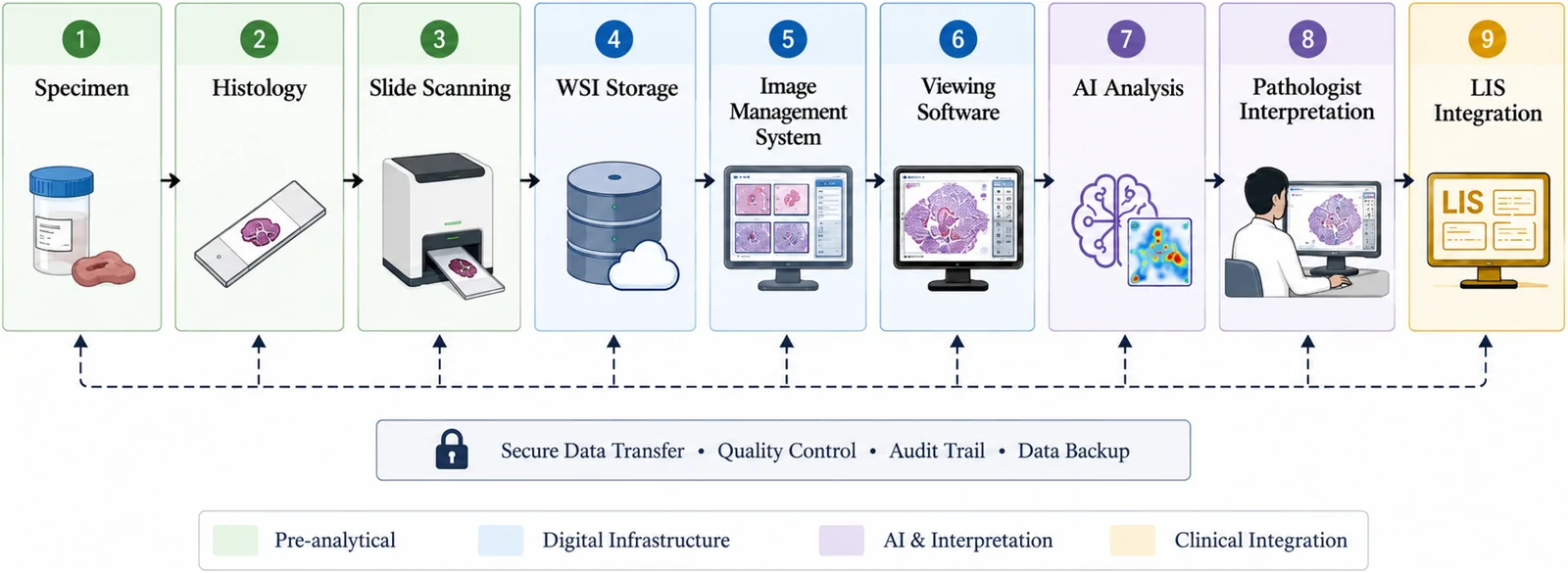
Components of the digital pathology workflow. Created in bioRender and ChatGPT (OpenAI, GPT-5.5).

### Whole-slide imaging

Over the past decade, advances in slide scanner, computing power, data storage, and network infrastructure have transformed digital pathology from a research tool into a FDA-approved platform for primary diagnosis.^7^ WSI systems digitize glass slides by acquiring thousands of high-resolution image tiles, which are subsequently stitched together to generate a seamless digital representation of the entire specimen.^8^, ^9^ These digital slides can then be viewed and analyzed using special image management and viewing software.

The imaging modality used during slide acquisition depends on the specimen type and staining protocol. Brightfield imaging is the standard approach for routine histopathology, including H&E, IHC, and special stains. In brightfield scanning, transmitted light passes through the tissue section to capture morphological and architectural details. Most clinical WSI systems scan brightfield slides at 20× or 40× objective magnification.^10^

In contrast, fluorescence imaging is used for applications such as fluorescence in situ hybridization, multiplex immunofluorescence, and quantitative biomarker analysis. Fluorescence scanners excite fluorophore-labeled targets with specific wavelengths of light and detect the emitted light, enabling visualization of nucleic acids, proteins, and other molecular markers.^11^ Similar objective magnifications (typically 20×–40×) are employed. Researchers have developed dual-mode scanners, including the Aperio FL,^12^ OptraSCAN OS-FLi,^13^ and SLIDEVIEW VS200,^14^ which integrate both brightfield and fluorescence imaging within a single platform, allowing labs to image multiple specimen types at the same time.

WSIs are stored in pyramidal multiresolution image formats that allow efficient viewing at multiple zoom levels without loading the entire image into memory. Common formats include pyramidal TIFF,^15^ DICOM-WSI,^16^ and proprietary vendor-specific formats (e.g., SVS, NDPI, and MRXS).^17^ These file formats support high bit depths, multiple imaging channels, image pyramids, and embedded metadata describing acquisition parameters and specimen information.^18^

Because digital pathology generates extremely large datasets, image compression and storage strategies are essential components of WSI infrastructure. Lossless compression preserves all pixel information and is generally preferred for research applications and AI model development.^19^ In contrast, visually lossless or lossy compression substantially reduces storage requirements and network bandwidth, making it practical for routine clinical workflows when diagnostic image quality is maintained.^20^

Storage demands vary considerably according to imaging modality, magnification, bit depth, tissue size, and number of fluorescence channels. A typical brightfield slide scanned at 20× occupies approximately 1–2 GB,^21^ whereas a multiplex fluorescence slide acquired at 40× with multiple channels may require 5–10 GB or more.

Due to the large size of WSIs, many institutions utilize cloud-based archival solutions to accommodate long-term retention.^22^ Picture archiving and communication systems serve as the central platform for storing, retrieving, managing, and displaying high-resolution WSIs.^23^ In addition, high bandwidth network connections are essential to minimize latency during image transfer and remote viewing.

## Artificial intelligence in digital pathology

### AI terminology

AI is a rapidly evolving field that has transformed technologies across numerous industries. AI encompasses a broad range of computational approaches, including machine learning and generative AI.^24^ In machine learning, algorithms learn patterns from data and generate predictions or decisions without being explicitly programmed. Depending on how the model learns, machine learning approaches are generally categorized as supervised, unsupervised, or reinforcement learning.^24^ Machine learning can be found in many applications we use in our lives, including spam filtering, recommendations for streaming platforms, fraud detection, and image recognition.

Machine learning can be further divided into traditional (conventional) machine learning and deep learning.^25^ Traditional machine learning relies on handcrafted feature extraction and statistical learning algorithms, whereas deep learning uses multilayer neural networks to automatically learn hierarchical representations from data. Common traditional machine learning algorithms include logistic regression, support vector machines, random forests, decision trees, and k-nearest neighbors.^26^ In contrast, deep learning encompasses diverse architectures, including convolutional neural networks (CNNs) such as ResNet, DenseNet, EfficientNet, Inception, and U-Net, vision transformers, and multimodal transformer-based models (Fig. 3).^26^

**Fig. 3:**
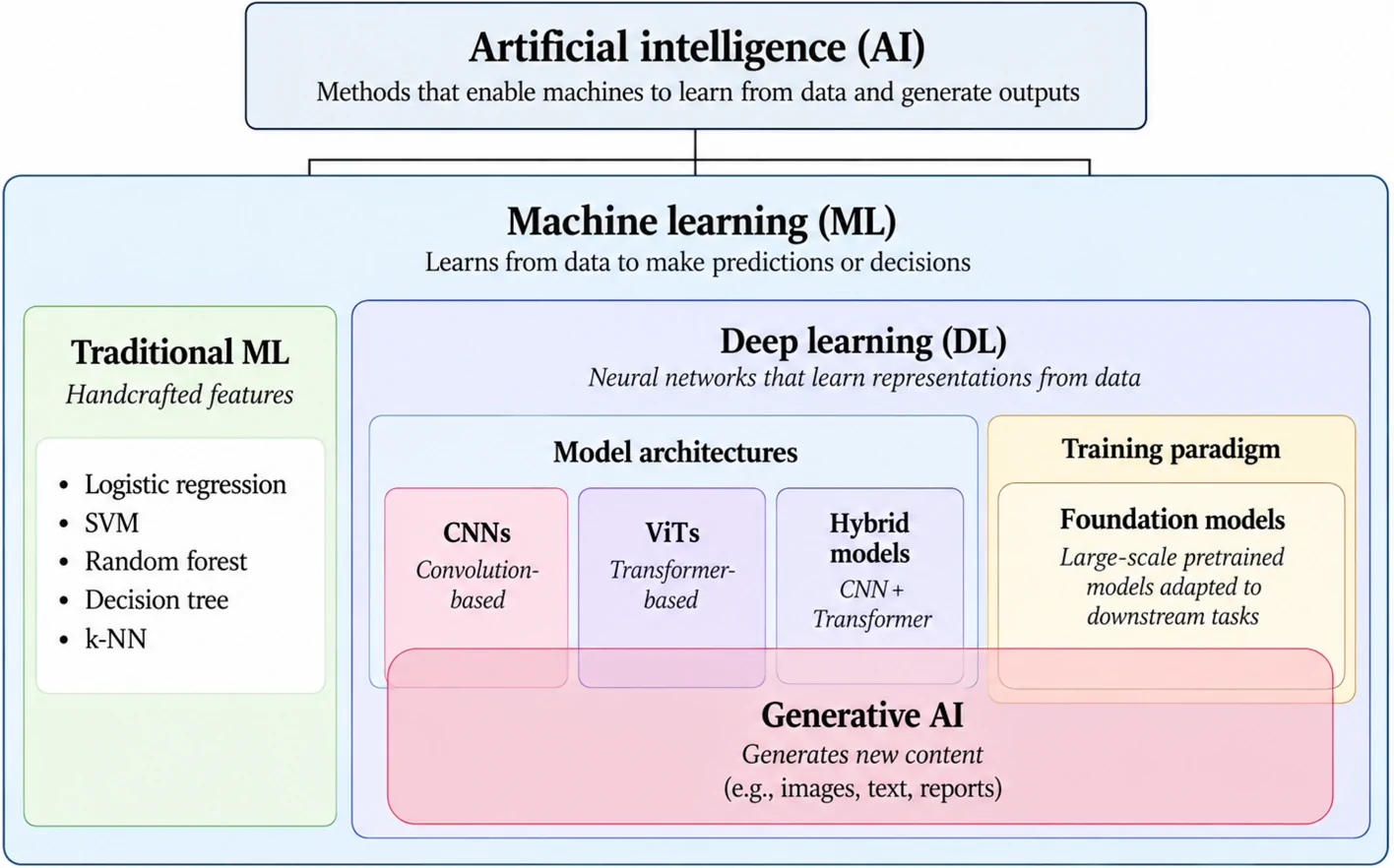
Hierarchical overview of AI technologies relevant to digital pathology. Created in bioRender and ChatGPT (OpenAI, GPT-5.5).

Generative AI, on the other hand, focuses on creating new content (e.g., images, text, music, etc.) that are original and unique. More recently, large language models (LLMs), including GPT, Gemini, Claude, and Llama, have greatly expanded the capabilities of generative AI and are now widely used for text generation, coding assistance, image creation, and scientific writing; many contemporary generative AI systems are themselves based on deep-learning architectures.^26^

Foundation models represent a complementary paradigm defined primarily by large-scale pretraining on broad datasets and subsequent adaptation to multiple downstream tasks, rather than by a single neural-network architecture.^26^ Thus, deep learning, generative AI, and foundation models are overlapping concepts that describe different aspects of AI systems, including their architecture, function, and training paradigm.

### AI models: Feature-engineering approaches to automated deep-learning frameworks

The digitization of histopathological slides has facilitated the application of increasingly sophisticated machine learning algorithms for image analysis. AI models range from conventional feature-engineering approaches, which involve manually defining or extracting measurable image characteristics such as cell size, cell shape, nuclear morphology, texture, color, and architecture, that an AI model uses for prediction, to fully automated deep-learning frameworks, where the AI model automatically learns relevant features directly from raw image data.^27^ There are four approaches, and in each approach, the AI model generates predictions that are evaluated against ground-truth labels derived from expert pathologist annotations (Fig. 4).

**Fig. 4:**
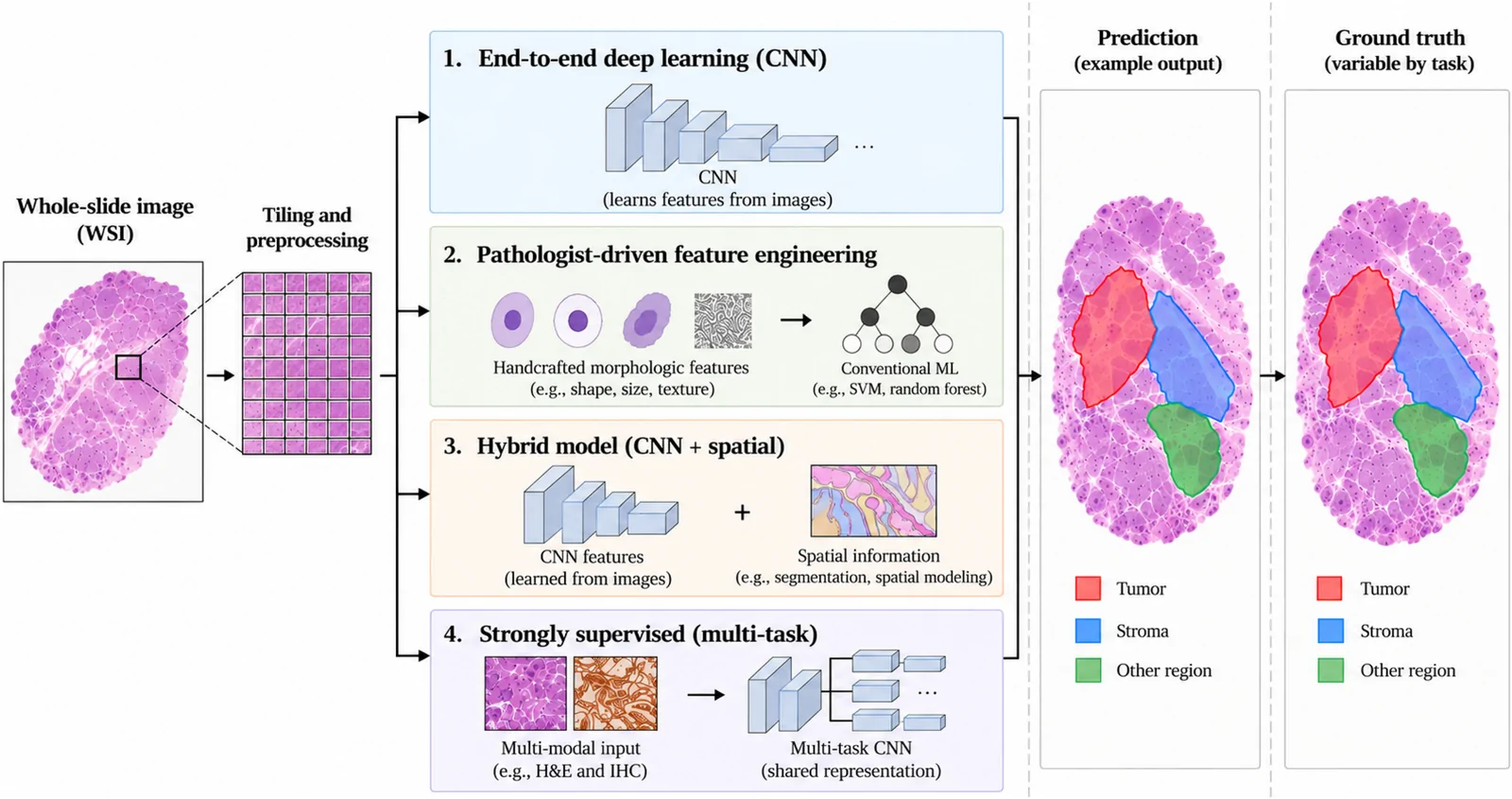
Overview of representative AI approaches for WSI analysis in computational pathology. Created in bioRender and ChatGPT (OpenAI, GPT-5.5).

The bottom-up approach relies entirely on deep learning, typically CNNs, to learn hierarchical feature representations directly from image tiles without manual feature engineering.^28^ During training of the AI model, early convolutional layers capture low-level image features, including edges, color gradients, and textures, whereas deeper layers learn increasingly complex cellular morphologies, tissue architecture, and higher-order spatial patterns. The learned feature representations are subsequently used to generate slide-level predictions.

In pathologist-driven approaches, diagnostically relevant morphological features are predefined based on domain expertise.^29^ These handcrafted features may include quantitative measurements of nuclear size, cellular morphology, tissue texture, glandular architecture, and spatial organization. The extracted measurements are summarized into feature vectors that serve as input to conventional machine learning algorithms, which subsequently generate predictions for comparison with the corresponding ground-truth labels.

Hybrid approaches combine deep learning with conventional image analysis methods to leverage the advantages of both paradigms.^30^ CNNs are initially employed to perform semantic or instance segmentation, generating pixel- or object-level maps that identify tissue components such as nuclei, cytoplasm, tumor epithelium, and stromal regions. Quantitative morphological and spatial features are then computationally extracted from the segmented structures and incorporated into downstream predictive models. This strategy integrates automated feature learning with interpretable feature engineering to improve predictive performance.

Finally, strongly supervised approaches utilize detailed annotations, including pixel-, cell-, or tile-level labels, during model training.^31^ Before analysis, WSIs typically undergo standardized preprocessing procedures, such as stain normalization, image registration, artifact removal, and tiling, to reduce technical variability. The availability of fine-grained annotations enables CNNs to learn precise morphological characteristics and tissue boundaries, thereby improving segmentation and classification accuracy.

Altogether, these approaches represent a progression from manually engineered feature-based models to fully automated deep-learning frameworks. This reflects the increasing complexity, annotation requirements, and predictive capabilities of contemporary computational pathology (Fig. 4).

### Multimodal AI

In the single-modality approach, pathologists interpret histopathological slides, radiological images, genomic data, and clinical information independently. In contrast, multimodal AI integrates information from multiple data sources to generate a more comprehensive representation of disease (Fig. 5). Depending on the model architecture, multimodal information can be integrated through early fusion, in which features from different modalities are combined before prediction; intermediate fusion, in which modality-specific representations interact within a shared latent space; or late fusion, in which predictions generated from individual modalities are combined.^32^ By capturing complementary information across data types, multimodal AI may overcome some limitations of image-only models and enable more comprehensive characterization of disease biology.

**Fig. 5:**
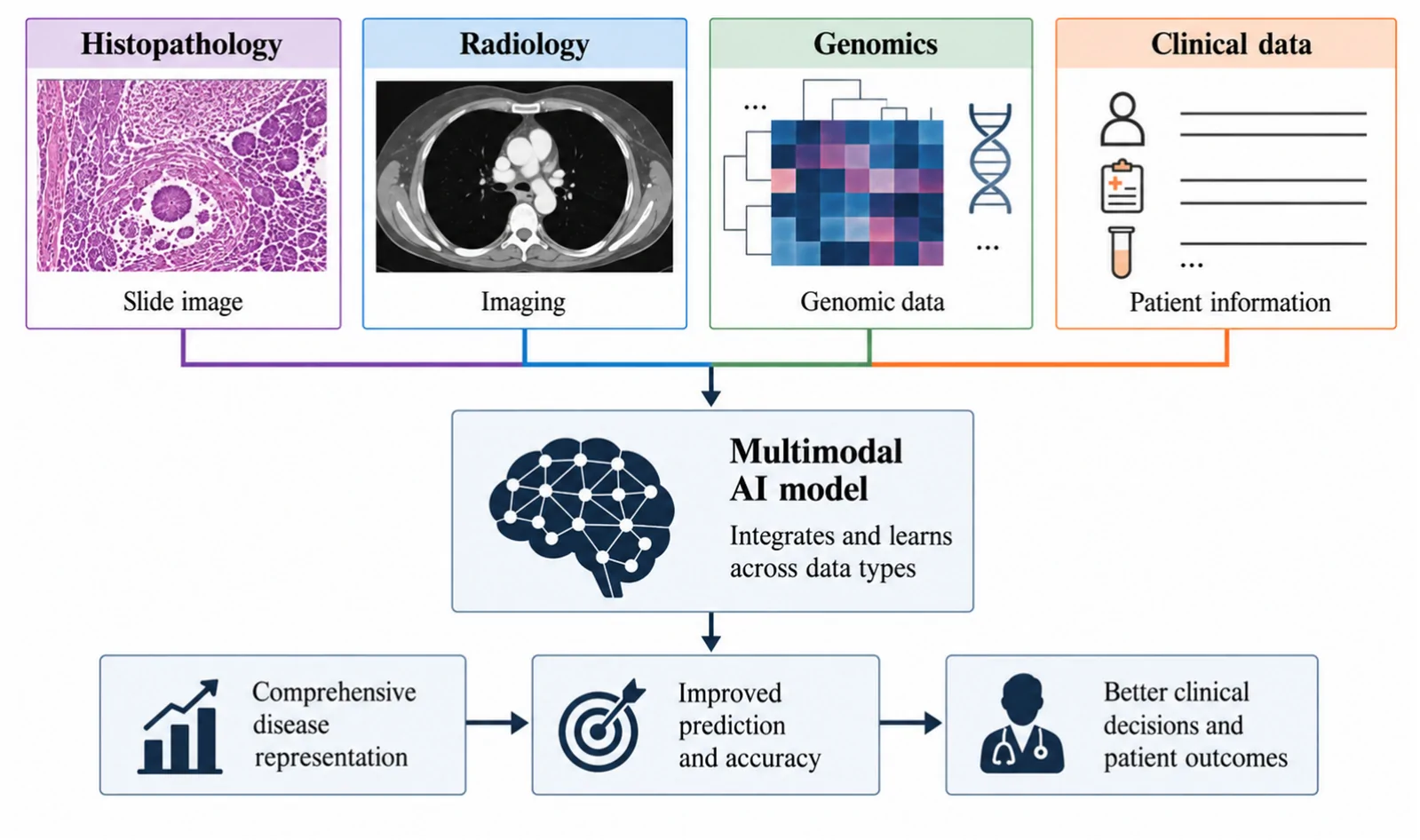
Multimodal AI in digital pathology. Created in bioRender and ChatGPT (OpenAI, GPT-5.5).

A multimodal AI application is histology–genomic integration. In this approach, morphological features extracted from WSIs are combined with genomic, transcriptomic, or other molecular data to identify associations between tissue morphology and tumor biology.^33^ These models predict clinically relevant biomarkers and molecular alterations, identify prognostic signatures, classify molecular subtypes, and improve risk stratification. Histology provides spatial and morphological information that may complement molecular measurements, whereas genomic and transcriptomic data provide information about tumor biology that cannot be directly observed through conventional microscopy. Consequently, integrating these modalities may facilitate the development of more comprehensive biomarkers and support precision oncology. However, these approaches require accurately matched histological and molecular datasets, and differences in sequencing platforms, tissue processing, patient populations, and institutional practices can introduce batch effects and domain shifts that may limit generalizability.

Another emerging application is three-dimensional (3D) computational pathology, in which AI models analyze reconstructed 3D tissue architecture rather than conventional two-dimensional (2D) histological sections.^27^ By capturing the spatial organization of cells, glands, vasculature, and the tumor microenvironment across multiple tissue planes, 3D computational pathology may provide information that is not fully captured by individual 2D sections. This approach may improve characterization of tumor invasion, spatial heterogeneity, cell–cell interactions, and treatment response, whereas potentially enabling more accurate assessment of complex tissue architecture. Nevertheless, widespread clinical implementation remains challenging because 3D reconstruction requires specialized imaging or tissue-processing approaches, substantial computational resources, and standardized methods for image acquisition, registration, and analysis.

More recently, multimodal LLMs have been introduced into pathology by integrating visual information from pathology images with natural-language representations. For example, PathChat is a general-purpose multimodal AI assistant that integrates pathology images with natural language interaction to perform a range of pathology-related tasks. In its initial evaluation, PathChat demonstrated strong performance on multiple-choice diagnostic questions spanning diverse tissue types and disease entities.^34^ Beyond question answering, image-language models may ultimately facilitate interactive case analysis, image-based retrieval, pathology report generation, and integration of histopathological findings with clinical context. However, these systems can produce plausible but incorrect responses, and their performance may vary according to disease type, image quality, clinical context, and the patient populations represented in their training data. Therefore, prospective clinical validation, transparent evaluation of failure modes, and appropriate human oversight will be necessary before multimodal foundation models can be reliably incorporated into routine diagnostic workflows.

Despite their potential, multimodal AI systems introduce additional challenges compared with single-modality models. Multimodal datasets require accurate linkage of images with corresponding clinical, molecular, or textual information, yet not all patients have the same types or completeness of data. Differences in data formats, acquisition protocols, institutional practices, and patient populations can further introduce domain shifts and batch effects. In addition, the integration of sensitive clinical and genomic information raises important privacy and governance considerations, whereas increasingly large multimodal models may require substantial computational and energy resources. Addressing these challenges through standardized data structures, multi-institutional validation, privacy-preserving data-sharing strategies, and robust evaluation across diverse populations will be essential for translating multimodal AI from research applications into clinically reliable tools.

## Clinical applications

Before discussing the clinical applications of AI in digital pathology, it is helpful to define the major categories of AI tasks: classification, detection, segmentation, prediction, and quantification.^29^ Although these tasks often overlap within a single workflow, each addresses a distinct aspect of histopathological image analysis (Fig. 6).

**Fig. 6:**
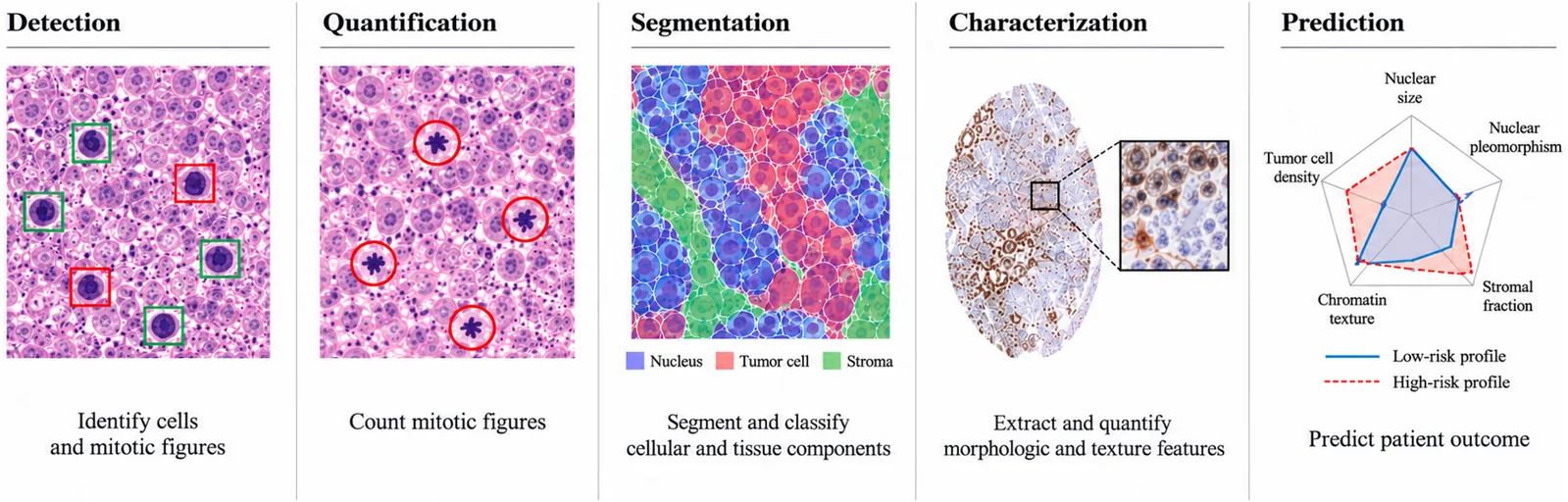
Representative applications of AI in computational pathology. Created in bioRender and ChatGPT (OpenAI, GPT-5.5).

Classification assigns an image, tissue region, or WSI to a predefined category. In digital pathology, classification models are commonly used for tumor subtype identification, tumor grading, assessment of treatment response, determination of the primary site of metastatic tumors, and distinction between benign and malignant lesions.^35^ Classification algorithms may also predict diagnostically and clinically relevant biomarkers directly from H&E-stained slides, including HER2,^36^ PD-L1,^37^ microsatellite instability,^38^ EGFR,^39^ IDH,^40^ MGMT,^41^ estrogen receptor,^42^ progesterone receptor,^43^ and Ki-67 status,^44^ providing complementary information for prognosis and therapeutic decision-making.

Detection focuses on identifying the presence and location of specific objects or abnormalities within a histopathological image. Examples include detecting tumor cells, metastatic deposits in lymph nodes, mitotic figures, microorganisms, or other diagnostically significant features while distinguishing them from normal tissue.

Segmentation involves delineating the precise boundaries of structures or regions of interest at the pixel level. This task is essential for identifying tumor margins, segmenting nuclei, glands, stromal regions, lymphocytes, blood vessels, or metastatic foci, thereby enabling detailed morphological analysis and quantitative assessment.

Prediction uses morphological features extracted from WSIs to infer clinically meaningful outcomes beyond visual diagnosis. AI models have demonstrated the ability to predict patient prognosis, recurrence risk, response to chemotherapy or immunotherapy, and underlying molecular alterations directly from routine H&E slides, supporting precision oncology.

Quantification measures the abundance or proportion of histological features within a tissue sample. Common applications include counting mitotic figures, tumor-infiltrating lymphocytes, positive IHC cells, and nuclei, as well as calculating tumor cellularity, biomarker expression, and other quantitative metrics. Automated quantification improves reproducibility and reduces the interobserver variability associated with manual assessment.

These five task categories form the foundation of most AI applications in digital pathology. In clinical practice, a single AI system frequently combines multiple tasks. This includes detecting tumor regions, segmenting individual nuclei, quantifying biomarker-positive cells, classifying tumor subtype, and predicting molecular alterations or patient outcomes from the same WSI.

## Current implementation in US medical centers

Since the introduction of WSI technology in the early 2000s, digital pathology has evolved rapidly through advances in scanner hardware, IMSs, and AI. Successive generations of WSI scanners have demonstrated substantial improvements in scan speed, throughput, image quality, slide capacity, and workflow efficiency.^45^

More specifically in the USA, a major milestone occurred in 2017, when the US FDA approved the Philips IntelliSite Pathology Solution as the first WSI system for primary diagnosis.^46^ This approval enabled pathologists to review and interpret digitized histopathological slides instead of relying exclusively on conventional light microscopy, paving the way for broader clinical adoption of digital pathology.

Since then, several commercial digital pathology platforms have become integral components of modern pathology workflows. Philips IntelliSite provides an end-to-end solution that combines high-resolution slide scanners, image management software, and digital slide viewers for routine clinical diagnosis. Leica Aperio is among the most widely adopted platforms in both clinical practice and research. This offers high-throughput scanners and integrated image management for primary diagnosis, education, telepathology, and AI-assisted image analysis.^47^ Roche NAVIFY Digital Pathology combines slide scanning with an enterprise image management platform that supports case sharing, LIS integration, and deployment of AI applications within routine diagnostic workflows.^48^ Hamamatsu NanoZoomer scanners are recognized for their high image quality, rapid scanning speed, and support for both brightfield and fluorescence imaging, making them widely used in academic research, pharmaceutical development, and an increasing number of clinical labs.^49^ Despite differences in hardware specifications, software ecosystems, and regulatory approvals, these systems all convert glass slides into high-quality WSIs for archival, remote review, and analysis computationally.

To ensure reliable implementation, professional organizations have established technical and operational recommendations for WSI systems. The College of American Pathologists (CAP) recommends that scanners support features such as high-throughput image acquisition, appropriate scanning magnification, *Z*-stacking, and extended depth of field imaging when clinically indicated.^50^ In addition, labs operating under the Clinical Laboratory Improvement Amendments (CLIA) must ensure that digital pathology workflows meet established standards for personnel qualifications, test validation, quality control, and quality assurance.^51^ In the USA, the Centers for Medicare & Medicaid Services (CMS) also plays an important role in the implementation of digital pathology through oversight of CLIA.^52^ CMS requires labs performing digital pathology for patient care to comply with CLIA regulations. In 2023, new Current Procedural Terminology codes for digital pathology image acquisition and digitization became effective, providing a standardized mechanism for reporting WSI scanning services.

In the USA, the FDA has cleared an expanding number of WSI systems for primary diagnosis of formalin-fixed paraffin-embedded surgical pathology specimens through the 510(k) pathway.^53^ Recent clearances include platforms from multiple major manufacturers, reflecting the continued expansion of digital pathology into clinical practice. Continued regulatory approvals, together with advances in scanner performance and AI-assisted image analysis, have accelerated the adoption of digital pathology across a wide range of practice settings. Although academic medical centers were early adopters and often maintain mature digital pathology infrastructures, community hospitals are increasingly implementing WSI systems as costs decline, workflows become more efficient, and regulatory guidance continues to mature.

The successful implementation of digital pathology at Memorial Sloan Kettering Cancer Center (MSKCC) illustrates how large institutions have transitioned from conventional microscopy to fully digital workflows. MSK adopted a phased implementation strategy that enabled gradual expansion of WSI while simultaneously developing the infrastructure required for enterprise-scale digital pathology. As part of this transition, the institution performed validation studies demonstrating that diagnoses rendered using WSI were equivalent to those made with conventional light microscopy, supporting the clinical adoption of digital pathology for primary diagnosis.^54^

Mayo Clinic provides another example of enterprise-level implementation: its digital pathology program has progressively expanded across its Rochester, Arizona, and Florida sites and the Mayo Clinic Health System, with digital workflows integrated into clinical practice and pathology consultation. More recently, Mayo Clinic has reported integration of WSIs with case information, EHRs, LISs, and AI-enabled tools, illustrating the evolution from simple slide digitization toward an integrated computational pathology ecosystem.^55^

Beyond large academic centers, digital pathology adoption has expanded steadily into community hospitals and regional health systems. Historically, implementation in community practice was limited by the high cost of scanners and information technology infrastructure, large image storage requirements, and concerns regarding workflow integration and reimbursement. However, improvements in scanner performance, cloud-based image management, regulatory guidance, and interoperability with LIS have made digital pathology increasingly accessible. In community settings, WSI is commonly used for primary diagnosis, remote consultation, telepathology, quality assurance, and subspecialty referrals, allowing smaller labs to obtain expert opinions without transporting glass slides.

## Challenges

Despite its rapid adoption and expanding clinical applications, digital pathology faces several technical, financial, operational, and regulatory challenges that must be addressed to enable widespread implementation. These challenges extend beyond the digitization of histopathological slides and include computational infrastructure, model generalizability, interpretability, data governance, health equity, and clinical accountability.

### Computational and infrastructure challenges

Computationally, WSIs generated by high-resolution scanners can contain several gigapixels, creating substantial demands for image storage, data transfer, and computational processing. Direct analysis of an entire WSI is often impractical because of its large size and memory requirements. Consequently, WSIs are commonly divided into smaller image patches that can be processed individually. Although patch-based approaches reduce computational demands, they may also limit the model's ability to capture spatial relationships and global tissue architecture across the entire slide. Hierarchical- and attention-based approaches have therefore been investigated to incorporate information across different spatial scales while maintaining computational feasibility.^56^, ^57^

The computational demands of digital pathology extend beyond image analysis. Large-scale AI models, particularly pathology foundation and multimodal models, require substantial computational resources for training, fine-tuning, and inference. These requirements can create barriers for institutions without access to high-performance computing infrastructure and may complicate deployment in resource-limited clinical environments.

The large size of WSIs also creates substantial data storage and information technology challenges. A single digital slide can require hundreds of megabytes to several gigabytes of storage depending on tissue area, scanning magnification, image compression, and file format. Large pathology labs may therefore accumulate very large image repositories over time, requiring investment in on-premises or cloud-based storage, high-speed networks, backup systems, and cybersecurity infrastructure. Long-term storage, retrieval, and transfer of these datasets represent continuing operational and financial considerations.

### Cost and workflow integration

Beyond computational requirements, cost remains an important barrier to digital pathology adoption. Implementing a digital pathology workflow requires capital investment in WSI scanners, IMSs, servers or cloud infrastructure, networking, and integration with LIMS. Additional costs may include software licensing, maintenance, cybersecurity, regulatory compliance, personnel training, and technical support. These financial requirements may be particularly challenging for community hospitals and smaller pathology practices with limited capital and information technology resources.^58^

Scanner throughput and workflow efficiency are also important considerations. Labs processing large specimen volumes require scanners capable of rapidly digitizing slides while maintaining consistent image quality. Commercial WSI platforms differ in slide capacity, scanning speed, autofocus performance, image quality, scan failure rates, and integration with existing lab workflows. Institutions must therefore consider scanner performance in relation to anticipated workload, staffing, turnaround-time requirements, and broader lab operations.^59^

### Generalizability, domain shift, and external validation

A major challenge for AI in digital pathology is generalizability, defined as the ability of a model to maintain its performance when applied to data from institutions or clinical settings that differ from those represented in the training dataset. Domain shift occurs when the distribution of deployment data differs systematically from that of the training data. In digital pathology, such differences may arise from variations in staining protocols, tissue fixation and processing, section thickness, scanners, imaging settings, magnification, specimen preparation, or patient populations.^60^

These technical and clinical differences can introduce institution- or batch-specific features that AI models may inadvertently learn instead of disease-relevant morphological characteristics.^61^ This problem can be particularly pronounced when technical characteristics are correlated with disease labels or patient populations in the training dataset. As a result, a model may demonstrate strong performance during internal validation but experience a decline in performance when evaluated on data from another institution with different data-generating conditions.

Multimodal and foundation-model approaches face additional challenges related to missing modalities.^62^ Not all patients undergo the same histological, IHC, molecular, radiological, or clinical assessments, and some modalities may be unavailable because of differences in clinical practice, specimen quality, acquisition costs, or incomplete records. Models trained primarily on complete multimodal datasets may therefore perform poorly when one or more modalities are unavailable. Approaches such as modality-masked training and computational imputation or reconstruction of missing modalities may improve robustness to incomplete data, but reconstructed information can introduce additional uncertainty if it does not accurately represent the underlying biology.

Data overlap between pretraining and evaluation datasets represents another concern. If the same patients, specimens, slides, or closely related cases are represented in both model development and evaluation datasets, information leakage can lead to artificially inflated estimates of model performance. This concern is particularly relevant for large foundation models trained on heterogeneous datasets assembled from multiple sources. Independent, patient-level separation of training, pretraining, validation, and test datasets is therefore important when assessing model performance.

These limitations underscore the importance of external, multi-institutional validation using independent datasets that reflect the diversity of clinical practice. Reporting performance across institutions, scanners, patient populations, disease subtypes, and relevant demographic groups can provide a more realistic assessment of whether a model has learned robust disease-related features rather than institution-specific characteristics.

### Interpretability and clinical trust

Interpretability and explainability remain important challenges for the clinical adoption of AI in digital pathology, particularly because many deep-learning models function as “black boxes,” in which the relationship between input features and the final prediction is not readily interpretable by human users.^63^ Visualization techniques such as attention maps and gradient-weighted class activation mapping (Grad-CAM) can provide post hoc evidence about model behavior by identifying image regions that contribute to a prediction.^64^, ^65^ These approaches may allow pathologists to assess whether a model is focusing on biologically and morphologically plausible features.

For example, if an AI model predicts a high-risk tumor and an interpretability map highlights a region containing poorly differentiated tumor cells, a pathologist can assess whether the highlighted morphology is consistent with the prediction. Conversely, attention directed toward tissue folds, necrotic debris, background tissue, or scanner artifacts may suggest that the model has learned a spurious correlation rather than disease-relevant features. Such visualization techniques can therefore support pathologist auditing of AI outputs and identification of potentially erroneous model behavior.

However, attention maps, Grad-CAM, and other post hoc methods do not make an AI model fully transparent or provide a complete representation of its underlying decision process. They should therefore be viewed as tools for auditing and hypothesis generation rather than definitive explanations of model reasoning. Interpretability approaches should complement, rather than replace, rigorous external validation, error analysis, and expert review.

### Data privacy and governance

Data privacy and governance represent additional challenges for the use of digital pathology data in AI research and clinical implementation. WSIs may contain protected health information within associated metadata and, in some cases, directly within scanned slide labels.^66^ De-identification procedures must therefore address both image content and associated metadata before datasets are used for research or shared across institutions.

Multi-institutional AI development may additionally require data-use or data-transfer agreements specifying permitted data uses, access controls, security requirements, data retention, and institutional responsibilities. These requirements can complicate the assembly of large, diverse datasets needed for robust AI development and validation. Privacy-preserving approaches, including federated learning, may facilitate collaborative model development while reducing the need to centrally transfer patient-level data, although they introduce their own technical and governance challenges.

### Bias and health equity

Bias and health equity also warrant greater consideration in digital pathology AI research. Training datasets are frequently derived from large academic or tertiary-care centers and may not adequately represent the demographic diversity, disease spectrum, and case mix encountered in community-based or resource-limited settings.^67^ Under-representation of particular populations may contribute to differences in model performance across demographic or clinical subgroups, whereas differences in referral patterns and disease prevalence may further limit generalizability.

Consequently, evaluation of digital pathology algorithms should incorporate diverse, multi-institutional cohorts and report performance across relevant demographic and clinical subgroups. Attention to differences in disease prevalence, specimen characteristics, and community case mix will be important to ensure that advances in digital pathology AI do not inadvertently contribute to disparities in diagnostic care.

### Medico-legal accountability

Liability and medico-legal accountability represent additional challenges associated with the clinical implementation of AI in digital pathology. When an AI system contributes to an incorrect diagnosis, responsibility may involve multiple stakeholders, including the pathologist, healthcare institution, and AI developer, depending on the circumstances surrounding development, validation, deployment, and clinical use. Because AI systems are generally implemented as decision-support tools rather than independent diagnostic authorities, questions remain regarding the appropriate allocation of responsibility when clinicians rely on erroneous algorithmic recommendations.^68^

Additional concerns include automation bias, inadequate documentation of AI-assisted decisions, software updates, and model failures resulting from distribution shifts between development and clinical environments. Clear standards for validation, monitoring, documentation, human oversight, and allocation of responsibility will therefore be important for the safe and accountable integration of AI into pathology practice.

### Clinical validation and the evolving role of the pathologist

Before digital pathology can be implemented for routine clinical diagnosis, labs must complete rigorous validation studies demonstrating that diagnoses rendered using digital slides are equivalent to those made using conventional light microscopy. CAP has published evidence-based recommendations and validation guideline statements to assist labs in establishing safe and reliable WSI workflows. These recommendations emphasize representative case selection, appropriate washout periods between digital and glass slide review, comprehensive documentation, and adequate pathologist training.^69^, ^70^

The implementation of AI introduces an additional layer of validation because labs must evaluate not only the reliability of the digital slide workflow but also the performance of the AI system within the intended clinical environment. Models should therefore be evaluated using representative clinical cases and monitored for performance changes following changes in scanners, staining protocols, software, patient populations, or other aspects of the clinical workflow.

AI is intended to augment rather than replace pathologists by improving efficiency, standardizing quantitative assessments, and assisting with selected diagnostic tasks. Successful implementation nevertheless requires pathologists to understand the capabilities and limitations of AI systems, critically evaluate model outputs, and recognize potential errors, biases, and distribution shifts. Ongoing education and training will therefore be important to ensure that AI functions as a decision-support tool while maintaining appropriate physician oversight and accountability for patient care.

### Computational cost and environmental sustainability

Finally, the increasing scale of pathology foundation and multimodal models raises concerns regarding computational expense, energy consumption, and environmental sustainability. Training and deploying increasingly large models can require substantial computational resources, particularly when models process high-resolution WSIs or multiple modalities simultaneously. These resource requirements may affect the feasibility of deploying advanced AI systems across institutions with limited computing infrastructure and may increase the environmental footprint of large-scale AI development. Future work should therefore consider computational efficiency, model size, hardware utilization, and energy requirements alongside predictive performance when developing and deploying AI systems in digital pathology.

## Future directions

Foundation models are expected to play an increasingly important role in the future of digital pathology. Unlike conventional AI models that are trained for a single task, foundation models are pretrained on millions of histopathology images using self-supervised learning, allowing them to learn general features of tissue architecture and cellular morphology. These pretrained models can then be adapted for a wide range of downstream applications, including disease diagnosis, biomarker prediction, prognosis, and slide retrieval, while requiring substantially fewer labeled training data.

The next major advancement will likely be the integration of foundation models with multimodal AI. Rather than analyzing WSIs alone, future systems will combine histopathology with pathology reports, radiological imaging, genomic data, and clinical information to generate a more comprehensive representation of disease. This approach has the potential to improve diagnostic accuracy, enhance prognostic prediction, and support more personalized treatment decisions, particularly for rare diseases where labeled data are limited.

Recent multimodal foundation models illustrate this emerging paradigm. The Transformer-based Pathology Image and Text Alignment Network, for example, jointly learns representations from WSIs and associated pathology reports, enabling transferable representations for a range of downstream pathology tasks.^71^ Similarly, the Multimodal transformer with Unified maSKed modeling was trained using large-scale, unpaired histopathology images and text and demonstrated strong performance across multiple patch- and slide-level benchmarks, including image–text retrieval, visual question answering, image classification, and molecular biomarker prediction.^72^ These approaches suggest that multimodal pretraining can produce generalizable representations that can be adapted across diverse pathology applications, although the extent to which they reduce or eliminate the need for task-specific fine-tuning varies by application.

The development of foundation models may also expand digital pathology beyond conventional classification and prediction toward generative and interactive applications. Emerging models are being designed to generate pathology-related text, retrieve morphologically similar cases, answer questions about histological images, and support natural-language interaction with clinicians. PRISM2, a multimodal slide-level foundation model, aligns histomorphological information with diagnostic reasoning and provides representations that can support prompt-based inference and downstream applications.^73^ As these capabilities mature, foundation models could potentially support multiple stages of the pathology workflow, including case triage, diagnostic assistance, report drafting, biomarker assessment, and retrieval of relevant prior cases. However, such applications should remain subject to pathologist oversight, with AI functioning as a decision-support technology rather than a replacement for expert interpretation.

Despite these advances, clinical translation remains a major challenge. Foundation models trained on large research datasets may not generalize across institutions because of differences in scanners, staining protocols, tissue preparation, patient populations, and lab workflows. In addition, the increasing scale and complexity of foundation models introduces challenges related to interpretability, computational requirements, data privacy, regulatory oversight, and reproducibility.^74^ Pathologist-in-the-loop evaluation will remain essential, with future studies assessing not only model performance but also diagnostic concordance, workflow efficiency, failure modes, and the impact of AI assistance on clinical decision-making.

Ultimately, the future of digital pathology is likely to involve a transition from isolated, task-specific AI algorithms toward integrated multimodal platforms that connect histomorphological information with molecular, radiological, and clinical data. Rather than automating individual diagnostic tasks in isolation, these systems could provide a more unified representation of disease and support a broader range of activities spanning diagnosis, prognostication, biomarker assessment, case retrieval, and treatment planning. Achieving this vision will require not only increasingly capable foundation models but also robust validation, standardized evaluation frameworks, interoperable data infrastructure, appropriate regulatory oversight, and sustained pathologist involvement in model development and clinical deployment.

## Consent statement

The authors have nothing to report.

## Ethics statement

The authors have nothing to report.

## Funding

Publication fees for this article were supported by a research fund from 10.13039/100008235Trinity School of Medicine, Trinity College Road, Ratho Mill, Kingstown, Saint Vincent and the Grenadines. The funding source had no role in the design, data collection, analysis, interpretation of data, writing of the manuscript, or the decision to submit the article for publication.

## Artificial intelligence disclosure

ChatGPT (OpenAI) was used to assist with rearranging sentences and paragraphs to improve the logical flow of the manuscript and to edit grammar and language. It was also used to assist with figure creation. ChatGPT was not used to generate scientific content, analyze data, interpret results, or draw conclusions. All content was critically reviewed, revised as necessary, and approved by the authors, who take full responsibility for the accuracy and integrity of the manuscript.

## Declaration of competing interest

The authors declare that they have no known competing financial interests or personal relationships that could have appeared to influence the work reported in this article.

## Data Availability

All data generated or analyzed are included in the article. No additional datasets were generated or analyzed beyond those presented in the article.
